# Protocol to detect spontaneous termination of yeast RNAPII transcription *in vitro*

**DOI:** 10.1016/j.xpro.2024.103369

**Published:** 2024-10-10

**Authors:** Zhong Han, Jesper Q. Svejstrup

**Affiliations:** 1 Department of Cellular and Molecular Medicine, Panum Institute, Blegdamsvej 3B, University of Copenhagen, 2200 Copenhagen N, Denmark

**Keywords:** molecular biology, gene expression, protein biochemistry

## Abstract

We present a protocol for the *in vitro* detection of spontaneous termination of yeast RNA polymerase II (RNAPII) transcription using bead-immobilized elongation complexes (ECs). We describe the steps for EC assembly, ligation to a long transcription template, and the *in vitro* elongation and termination reactions. Our protocol has proven successful for identifying spontaneous termination in the yeast CYC1 terminator.

For complete details on the use and execution of this protocol, please refer to Han et al.^1^

## Highlights

• Protocol for yeast RNAPII purification
• Steps for *in vitro* assembly of yeast RNAPII elongation complex
• Instructions for *in vitro* RNAPII transcription and termination assay

## Abstract (editor highlights)

Publisher’s note: Undertaking any experimental protocol requires adherence to local institutional guidelines for laboratory safety and ethics.

## Graphical abstract

**Figure:**
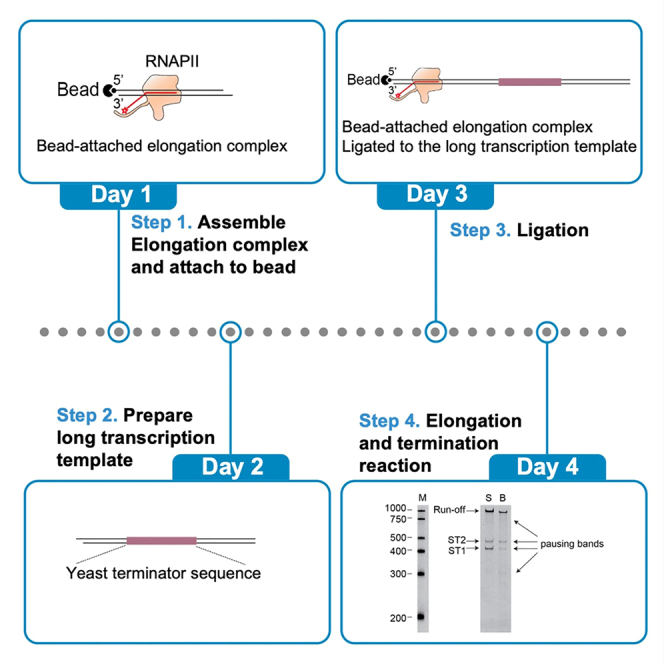

## Before you begin

The protocol below describes the specific steps for biochemically detecting spontaneous termination of yeast RNAPII transcription at *Saccharomyces cerevisiae* terminator sequences *in vitro*.

Before initiating the transcription assays, several key materials must be prepared. These include purified yeast RNAPII, long DNA oligonucleotides, and RNA markers. RNAPII and oligonucleotides are required to assemble the elongation complex, while RNA markers are needed to determine the size of the *in vitro* transcribed RNA.

### Yeast RNAPII purification

**Timing: 1 week**

1. Culture yeast, *Saccharomyces cerevisiae*, expressing Rpb3-flag (in a *Δprb1* strain, a protease-deficient yeast strain) in 10 L YPD media and harvest at OD_600_ 5–10.

***Note:*** Rpb3 is chosen for the flag tag in RNAPII due to its position in the enzyme's structure. The flag tag is exposed on the surface, making it accessible for purification purposes. Alternatively, tags on the Rpb1 subunits might also be worth trying.

2. Resuspend the pellet in 1/10 pellet volume with lysis buffer and make into frozen droplets by dispensing drop by drop directly into liquid nitrogen. Then break yeast cells by using a freezer mill (SPEX 6875D Freezer/Mill) with standard program: rate at 15 cycle per second for 6 cycles of 2 min grinding and 2 min cooling each.

***Note:*** The resulting frozen powder can be stored at −80°C for several years.

3. For purification (at 4°C unless otherwise noted), resuspend the frozen powder in an equal volume of cold lysis buffer. After resuspension, centrifuge for 1 h at 4 °C at 40000 rpm in a 45 Ti rotor and collect the supernatant.

**CRITICAL:** The purification step should be done at a low temperature (4°C) using a cold chamber or in a cold room.

4. Flag purification.
  a. Load supernatant onto Flag resin (Sigma, A2220) packed column (Bio-Rad, 7321010), typically 3 mL dry-bead volume per 100–200 mL of extract.
  b. Wash with 30 mL wash buffer 1.
  c. Wash with 30 mL wash buffer 2.
  d. Add 3 mL elution buffer and use a pipette to mix. Then cap the column and incubate the resuspended beads for 15 min with rotation. Collect the eluate and repeat this step four more times. Pool all the fractions to obtain approximately 15 mL flag elution.
5. MonoQ purification.
  a. Load the Flag elution onto a 1 mL Mono Q 5/50 GL column (Cytiva, 17–5166-01) by using a chromatography system, such as the ÄKTA Pure.
  b. Elute the target protein using a potassium acetate (KOAc) gradient between Buffer A and Buffer B at a flow rate of 0.5–1 mL/min over a volume of 10 mL. Collect the samples in 0.5 mL fractions by using a fraction collector, such as F9-R (Cytiva, 29011362).
  c. Perform SDS-PAGE analysis of the fractions containing proteins.

***Note:*** SDS will precipitate with potassium and co-precipitate with the purified protein. Load everything, including the precipitates, onto the SDS-PAGE.

6. Combine the fractions containing RNAPII, and then concentrate and exchange buffer against MonoQ buffer A using e.g., Vivapsin 20 concentrators MWCO 100 KD (Sigma, Z614661).

***Note:*** Add MonoQ buffer A to the pooled MonoQ elution (1–2 mL) to a total volume of around 15 mL. Concentrate to approximately 1–2 mL. Dilute it back to 15 mL with MonoQ buffer A and concentrate again. Repeat this process three times to achieve complete buffer exchange. The final protein concentration should be approximately 0.5 mg/mL (1 μM), with a total RNAPII amount of around 1 mg.

7. Add glycerol to a final concentration of 5%, aliquot 20 μL portions and snap freeze in liquid nitrogen, store at −80°C.

### Purification of long DNA oligonucleotides

**Timing: 2 days**

8. Design of DNA oligonucleotides to assemble an elongation complex.
  a. The 5′ end of the transcription template strand contains a phosphor modification to facilitate ligation.
  b. The 5′ end of the non-template strand has a biotin modification for attachment to streptavidin dynabeads.
  c. A BamHI site was designed at the RNAPII binding region. If the EC (elongation complex) is correctly assembled, it will protect the site from being digested by the BamHI enzyme.
9. Run the DNA oligonucleotides on a 10% polyacrylamide gel.
  a. Dissolve lyophilized oligonucleotides (purchased in desalt format from e.g., Integrated DNA Technologies (IDT)) in water to a concentration of 300 μM.
  b. Take 30 μL of the oligonucleotide solution and add 1/10 volume 50% glycerol.
  c. Load samples on the gel (7.2 cm × 8.6 cm, 10 well comb) and load bromophenol blue dye into a separate well. Run the gel at around 100 V in TBE until the bromophenol blue dye migrates to the bottom of the gel.

***Note:*** PAGE-purified DNA oligonucleotides could also be purchased directly from companies.

Several oligonucleotides may be loaded on the same gel for purification. Bromophenol blue dye (0.25% bromophenol blue, 5% glycerol) was homemade, but any commercial DNA loading dye containing bromophenol blue should work. In a 10% polyacrylamide gel, bromophenol blue migrates at the position of a 12-nt oligonucleotide.

10. Gel extraction.
  a. Expose the gel (without nucleic acid dye) under epi-blue light for 10 s (similar wavelength as SYBR Green, excitation at 472 nM, emission at 595 nM) using an Azure imaging system or similar (Azure biosystems).
    ***Note:*** UV shadowing is another option to view the bands without using dye.
  b. Print the image and cut out the oligonucleotide bands according to the image.
    ***Note:*** The oligonucleotide bands are often very faint; therefore, use photoshop to increase the contrast to better identify the location of the bands.
  c. Transfer the gel pieces to a 1.5 mL microtube.
  d. Add 300 μL TE buffer.
  e. Freeze for 10 min at −80°C or until solid, next quickly thaw at 50°C in a water bath (or a heat block with shaking) and then continue incubating at 50°C for 20 min.
  f. Repeat the freeze and thaw cycle.
  g. Spin briefly and carefully take 240 μL supernatant and transfer it to a new 1.5 mL microtube.
11. Ethanol precipitation.
  a. Add 14 μL 5 M NaCl, then add 750 μL 100% ethanol to precipitate the oligonucleotides.
  b. Mix thoroughly and spin at 18000 *g* at 4°C for 30 min.
    ***Note:*** Spinning immediately after mixing should recover more than 85% of the oligonucleotide. If a higher yield is required, precipitate oligonucleotides at −20°C overnight before spinning.
  c. Discard supernatant and add 800 μL 70% ethanol to wash the pellet.
  d. Spin at 18000 *g* at 4°C for 10 min and discard supernatant.
  e. Spin at 18000 *g* at 4°C for 1 min and discard the remaining supernatant.
  f. Dry the oligonucleotide pellets with the lid open for 10 min at room temperature.
  g. Resuspend oligonucleotides in 50 μL water.
12. Quantify the DNA concentration by a Nanodrop spectrophotometer and dilute to 10 μM with water.

### Production of fluorescently labeled RNA marker

**Timing: 2 h**

13. Prepare a T7 transcription reaction by using a MAXI script (AM1312) kit.

**Table:** 

Total reaction	20 (μL)
10X transcription buffer	2
10 mM ATP	1
10 mM UTP	1
10 mM GTP	1
10 mM CTP	1
5 mM Fluorescein-12-UTP	1
RNA marker templates, AM7782, 0.5 mg/mL	1
RNaseOUT	1
Enzyme mix	1
H_2_O	10

14. Mix and incubate at 37°C for 1 h, add 2 μL 0.5 M EDTA to stop the reaction, heat to 80°C for 5 min to denature the proteins. Store the RNA marker at −80°C. It should be stable for 6 months.

## Key resources table

**Table:** 

REAGENT or RESOURCE	SOURCE	IDENTIFIER
**Chemicals, peptides, and recombinant proteins**		

Sodium deoxycholate	Thermo Fisher Scientific	Cat # 89904
Complete EDTA-free protease inhibitor cocktail tablets	Sigma-Aldrich	Cat # 11873580001
3x FLAG peptide (DYKDDDDK-DYKDDDDK-DYKDDDDK)	Peptide Chemistry, The Francis Crick Institute, but any commercial 3x FLAG peptide should work.	N/A
Anti-FLAG M2 agarose	Sigma-Aldrich	Cat # A2220
Mono Q 5/50 GL	Cytiva	Cat # 17–5166-01
Vivaspin 20 centrifugal concentrator	Sigma-Aldrich	Cat #Z614661
BamHI-HF	NEB	Cat #R3136L
BsaI-HF	NEB	Cat #R3733L
Fast DNA ladder	NEB	Cat #N3238S
Q5 Hot Start high-fidelity DNA polymerase	NEB	Cat #M0494L
Dynabeads Myone Streptavidin T1	Thermo Fisher Scientific	Cat # 65602
Invitrogen 0.1–2 kb RNA ladder	Thermo Fisher Scientific	Cat # 11518766
RNA marker templates	Thermo Fisher Scientific	Cat # AM7782
Triton X-100	Thermo Fisher Scientific	Cat # HFH10
Heparin	Sigma-Aldrich	Cat # 3393
RNaseOUT	Thermo Fisher Scientific	Cat # 10777019
GlycoBlue coprecipitant	Thermo Fisher Scientific	Cat # AM9516
TBE-Urea gels, 6%	Thermo Fisher Scientific	Cat # EC68655BOX
Amicon Ultra-4 centrifugal filter unit	Merck Millipore	Cat # UFC8100
RNase OUT	Invitrogen	Cat # 10777-019
Fluorescein-12-UTP	Jena Bioscience	Cat # NU-821-FAMX

**Critical commercial assays**		

Pierce 660 nm Protein Assay Reagent	Thermo Fisher Scientific	Cat # 22660
MAXIscript T7 transcription kit	Thermo Fisher Scientific	Cat # AM1312

**Experimental models: Organisms/strains**		

Rpb3-flag::NAT, prb1Δ	Kind gift from John Diffley	N/A

**Oligonucleotides**		

oZH102, 5′ FAM labeled RNA	Integrated DNA Technologies	/56-FAM/UUUUUCGACCAGGA
oZH103, Template DNA strand for assembling elongation complex. 5′ phosphor modification to help ligation.	Integrated DNA Technologies	/5Phos/GTTGTGCAGGCCGGGTG CGGCCGCCCGTGTGGAGATGGG TGAGAGATGTTGAGGATCCTGGT CGTTTCCTATAGTTTGTTTCCTGA GTAAGTCTTCATCG
oZH104, Non-template strand for assembling elongation complex. 5′ biotin labeled to attach to streptavidin beads.	Integrated DNA Technologies	/5Biosg/CTAGCGATGAAGACTTAC TCAGAAACACGACTTAGGTAGAC GACCAGGATCCTCAACATCTCTC ACCCATCTCCACACGGGCGGCC GCACCCGGCCTGCA
oZH106, Fwd primer to PCR amplify long transcription template containing CYC1 terminator sequence. Contains BsaI cleavage site to produce sticky ends for ligation to assembled EC.	Integrated DNA Technologies	tactGGTCTCacaaccgttcgtcctcactctcttc
oZH39, Rev primer to PCR amplify long transcription template containing CYC1 terminator sequence.	Integrated DNA Technologies	GCTATGACCATGATTACGCCAAG

**Recombinant DNA**		

Plasmid containing CYC1 terminator sequence	Zhong et al.^1^	pZH20

**Software and algorithms**		

Adobe Photoshop	Adobe	https://www.adobe.com/au/products/photoshop.html
Adobe Illustrator	Adobe	https://www.adobe.com/uk/products/illustrator.html

**Other**		

Freezer/Mill	Ramcon	6875
ÄKTA Pure FPLC (or similar)	Cytiva	N/A
Fraction collector F9-R	Cytiva	29011362
Typhoon FLA 9500	Cytiva	Cat #: 29-0040-80
Azure 200	Azure Biosystems	https://azurebiosystems.com/azure-200/
DynaMag-2 Magnet	Invitrogen	Cat #: 12321D

## Materials and equipment

**Table:** Lysis buffer

Reagent	Final concentration	Amount
HEPES-NaOH (1 M) pH 7.6	50 mM	50 mL
ammonium sulfate (1 M)	400 mM	400 mL
MgSO4 (1 M)	10 mM	10 mL
EDTA (0.5 M)	1 mM	2 mL
Glycerol (50%)	10%	200 mL
ddH_2_O	N/A	338 mL
**Total**	**N/A**	**1000 mL**

Storage conditions: Store at 4°C for six months. Protease inhibitor should be added to the buffer immediately before use, after which the buffer can be stored at 4°C for up to 2 h.

**Table:** Wash buffer 1

Reagent	Final concentration	Amount
HEPES-NaOH (1 M) pH 7.6	50 mM	50 mL
Ammonium sulfate (1 M)	400 mM	400 mL
MgSO4 (1 M)	10 mM	10 mL
EDTA (0.5 M)	1 mM	2 mL
ddH_2_O	N/A	538 mL
**Total**	**N/A**	**1000 mL**

Storage conditions: Store at 4°C for six months.

**Table:** Wash buffer 2

Reagent	Final concentration	Amount
HEPES-NaOH (1 M), pH 7.6	50 mM	5 mL
NaCl (5 M)	500 mM	10 mL
EDTA (0.5 M)	1 mM	0.2 mL
Na-deoxycholate 10%	0.1%	1 mL
ddH_2_O	N/A	83.8 mL
**Total**	**N/A**	**100 mL**

Storage conditions: Store at 4°C for six months.

Na-deoxycholate stock solution should be protected from light.

**Table:** FLAG Elution buffer

Reagent	Final concentration	Amount
HEPES-NaOH (1 M), pH 7.6	50 mM	0.75 mL
KOAc (1 M)	110 mM	1.65 mL
EDTA (0.5 M)	1 mM	0.03 mL
FLAG peptide (5 mg/mL)	0.1 mg/mL	0.3 mL
ddH_2_O	N/A	12.27 mL
**Total**	**N/A**	**15 mL**

Storage conditions: Freshly prepared before elution step.

**Table:** MonoQ buffer A

Reagent	Final concentration	Amount
HEPES-NaOH (1 M), pH 7.6	50 mM	50 mL
KOAc (1 M)	110 mM	110 mL
EDTA (0.5 M)	1 mM	2 mL
MgOAc (1 M)	5 mM	5 mL
DTT (1 M)	10 mM	10 mL
ddH_2_O	N/A	823 mL
**Total**	**N/A**	**1000 mL**

Storage conditions: Freshly prepared before MonoQ purification.

### MonoQ buffer B

MonoQ buffer A with 2 M KOAc.

Storage conditions: Freshly prepared before MonoQ purification.

**Table:** Transcription buffer (TB)

Reagent	Final concentration	Amount
Tris-HCl (1 M), pH 7.5	20 mM	1 mL
NaCl (5 M)	100 mM	1 mL
MgCl2 (1 M)	8 mM	0.4 mL
ZnCl2 (10 mM)	10 μM	0.05 mL
DTT (1 M)	2 mM	0.1 mL
BSA (10 mg/mL)	0.1 mg/mL	0.5 mL
Glycerol (50%)	10%	10 mL
ddH_2_O	N/A	36.95 mL
**Total**	**N/A**	**50 mL**

Storage conditions: Store at −20°C for six months.

### TB_Triton

TB with 0.1% Triton X-100.

Storage conditions: Store at −20°C for six months.

### TB_1K

TB with 1 M NaCl.

Storage conditions: Store at −20°C for six months.

### TB_300

TB with 300 mM NaCl.

Storage conditions: Store at −20°C for six months.

### TB_hep

TB with 50 μg/mL heparin.

Storage conditions: Store at −20°C for six months.

### Urea loading buffer

8 M urea in 1 X TBE buffer.

Storage conditions: Store at room temperature for six months.

## Step-by-step method details

We provide a detailed step-by-step protocol for assembling the RNAPII elongation complex, followed by ligation to a long transcription template containing a spontaneous termination sequence, and conducting the transcription and termination assay.

### Assemble elongation complex

**Timing: 1 day**

**Figure 1:**
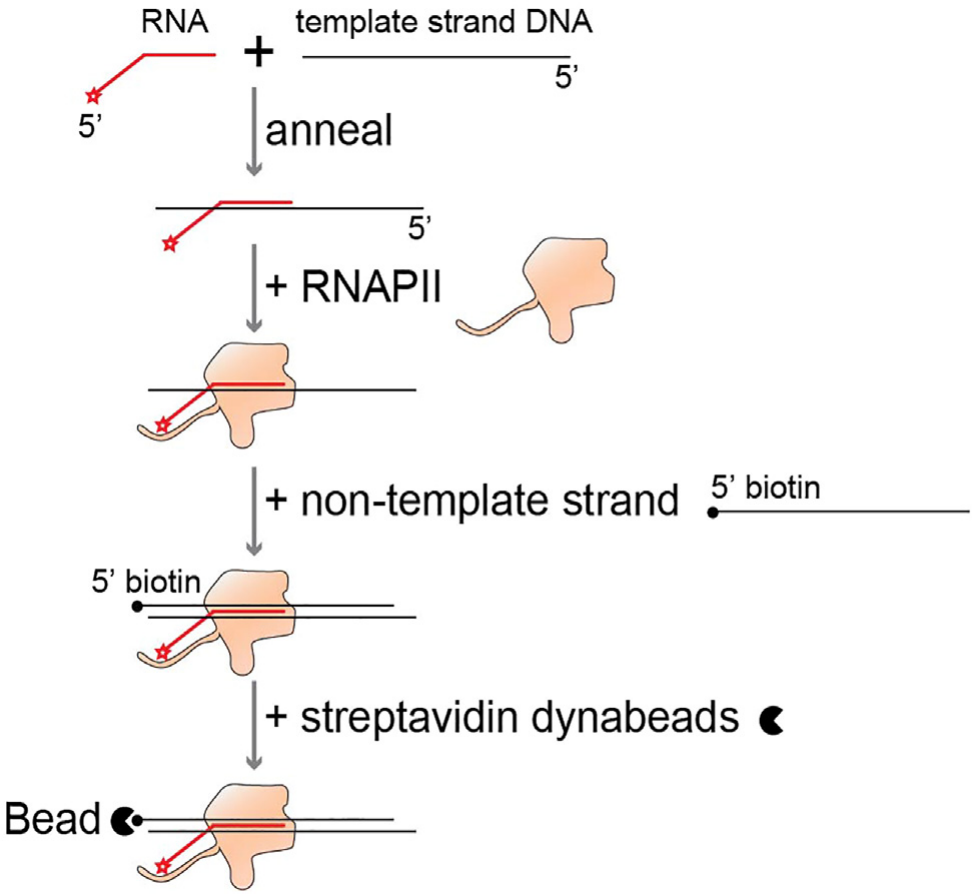
Schematic model depicting the process of assembling the RNAPII elongation complex

This step describes how to assemble elongation complexes and immobilize them on beads (Figure 1).

1. RNA:DNA annealing.
  a. Mix the RNA (oZH102, 5′ FAM labeled RNA) and the template DNA (oZH103, Template DNA strand) 1:1 ratio, each 5 μM, in 20 μL of 10 mM Tris-HCl pH 7.5, 50 mM NaCl.
  b. Annealing: Heat at 95°C for 2 min, then gradually cool by switching off the thermoblock until the temperature has dropped to 25°C.
  c. Store at −20°C.

**Pause point:** The annealed RNA-template DNA can be stored at −20°C until use. They should be stable for at least one year.

2. RNAPII-RNA-template DNA complex assembly.
  a. Thaw an aliquot of purified RNAPII on ice.
  b. Mix 5 μL of 1 μM purified RNAPII (dilute with MonoQ buffer A if needed) with 1 μL of 5 μM RNA-template DNA.
  c. Add 1 μL of 10 mg/mL BSA, and 1 μL 1 M ammonium sulfate.
  d. Incubate for 40 min at 30°C, shaking at 300 rpm.

**CRITICAL:** Adding BSA and ammonium sulfate in the assembly reaction improves assembly efficiency. Longer incubation time (40 min vs 10 min) and higher incubation temperature (30°C vs 25°C) also enhance efficiency.

***Note:*** Any remaining unused RNAPII can be snap-frozen and stored at −80°C. Up to 5 freeze-thaw cycles are acceptable for maintaining RNAPII activity.

3. Assembly of the elongation complex (EC) with the non-template DNA strand oZH104.
  a. Add 1 μL of 10 μM non-template DNA.
  b. Incubate for 15 min at 30°C, shaking at 300 rpm.
4. Immobilization of the ECs via the non-template DNA biotin-tag to streptavidin-magnetic dynabeads (MyOne streptavidin T1).
  a. Use 10–20 μL streptavidin T1 beads slurry for each assembly.
  b. Wash the beads three times with 200 μL TB_Triton.
  c. Mix the beads, in 5 μL TB_Triton, with the assembled EC.
  d. incubate for 30 min at 30°C, 1400 rpm (1 min ON, 4 min OFF).
5. Wash the assembled and bead-immobilized EC.
  a. Wash once with TB_Triton by adding 200 μL TB_Triton. Incubate for 1 min at 30°C with shaking at 1400 rpm. Separate the supernatant from the beads using a Dynabead magnet and discard the supernatant.
  b. Similarly, once with TB_1K.
  c. Similarly, once with TB_hep.
  d. Similarly, once with TB.

**CRITICAL:** Washing with transcription buffer containing high salt or heparin can substantially reduce contaminants, such as RNases.

6. Snap-freeze in liquid nitrogen, and store at −80°C.

**Pause point:** The assembled EC can be stored at −80°C and remains stable for at least 3 months. It can also withstand several rounds of freezing and thawing without significant loss of function.

### Ligation to a long transcription template

**Timing: 1 day**

**Figure 2:**
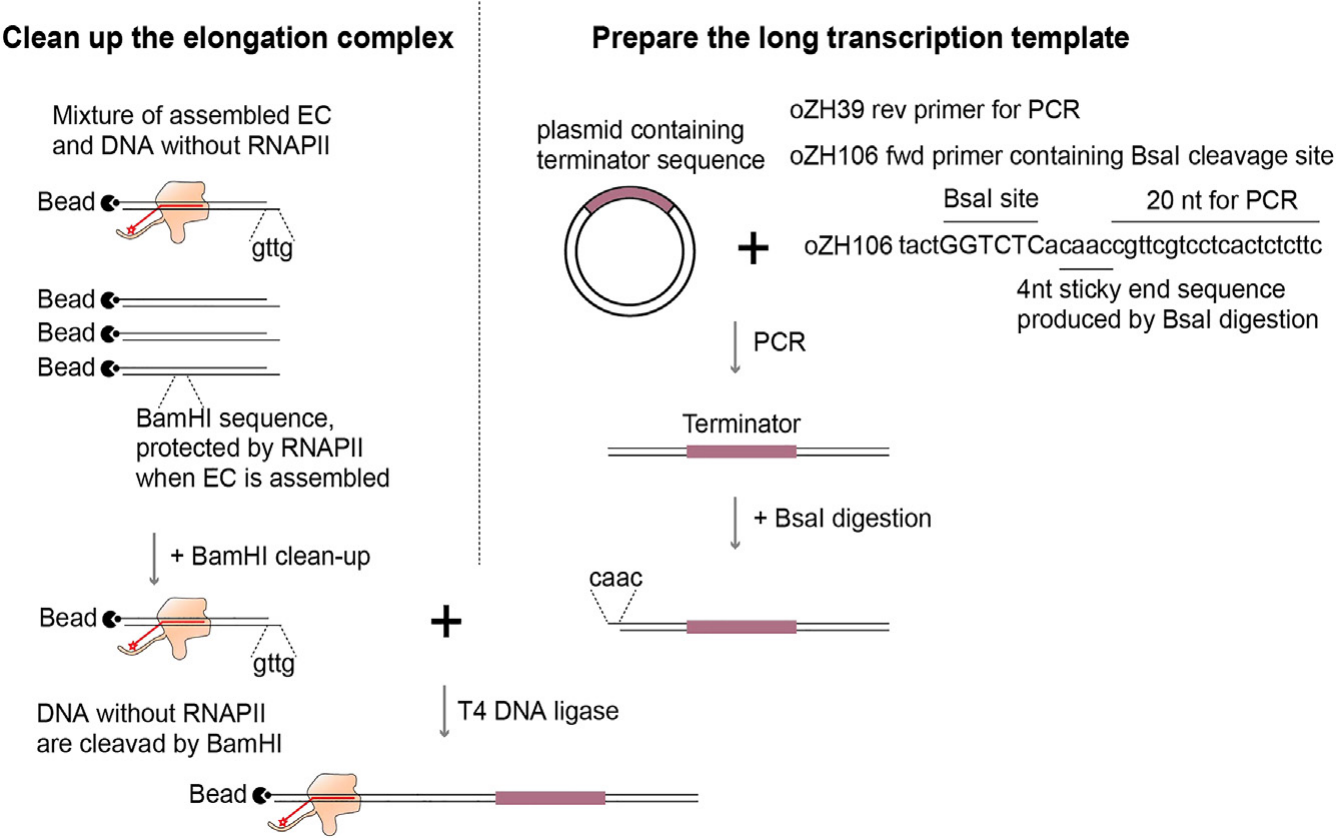
Schematic depicting the ligation of the RNAPII elongation complex to a long transcription template

This step describes how to ligate the assembled EC to a long transcription template (Figure 2).

7. Prepare a long transcription template.
  a. PCR amplify the CYC1 terminator from pZH20 by primer oZH106 and oZH39.
    ***Note:*** oZH106 contains a BsaI cleavage site to produce sticky ends for ligation to the assembled EC. Other *S. cerevisiae* terminator sequences can be found at Saccharomyces Genome Database (yeastgenome.org) and cloned into any desired plasmid.
  b. Agarose-gel purify the PCR fragment by any commercial kit.
  c. Digest the fragment with BsaI to produce a sticky end.
8. Ligate assembled EC with the long transcription template.
  a. Pretreat the assembled EC with BamHI to remove any DNA templates without RNAPII.
    **CRITICAL:** BamHI site is in the EC region. If EC is correctly assembled, then it will protect the site from being digested by the BamHI enzyme, and the gttg sites are still present to be ligated to the long transcription template.
  b. Prepare ligation mix. Assembled EC from 15 pmol starting material (which typically yields 1.5 pmol final product) is ligated to 3 pmol (approximately 1 μg) long DNA template using 4KU T4 DNA ligase.
  c. Incubate for 1 h at 22°C, occasional shaking at 1400 rpm (1 min ON, 4 min OFF).
9. Wash ligated EC using Dynabead magnet as in step 5.
  a. Once with TB_1K.
  b. Once with TB_hep.
  c. Once with TB.
10. Snap-freeze in liquid nitrogen and store at −80°C.

**Pause point:** The ligated EC can be stored at −80°C and remains stable for at least 3 months. It can also withstand several rounds of freezing and thawing without significant loss of function.

### *In vitro* elongation and termination reaction

**Timing: 1 day**

**Figure 3:**
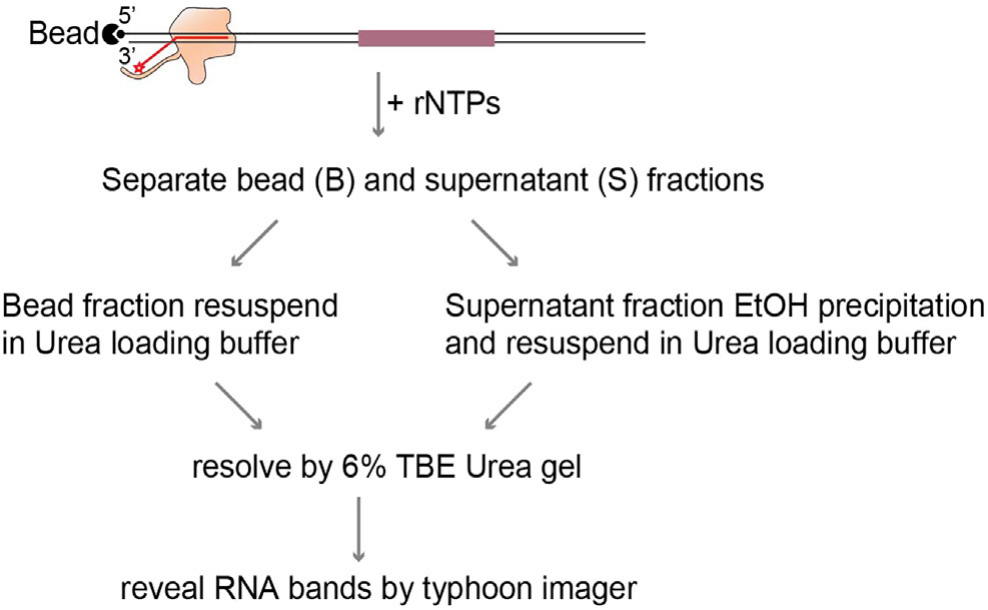
Schematic depicting the transcription reaction

This step describes how to perform the elongation and termination reaction, as well as how to analyze the results. After the transcription reaction, both the supernatant and bead-bound fractions should be loaded onto a TBE-Urea gel. The bead-bound fraction reveals the pausing sites of the elongation complex, while the supernatant fraction indicates where the elongation complex dissociates from the transcription template, releasing its RNA product (Figure 3).

11. Resuspend the ligated EC (bead-bound) with 18 μL TB_300, add 1 μL RNaseOUT.
12. Add 1 μL of 2 mM NTPs and mix by pipetting to initiate transcription elongation at 30°C for 10 min.
13. After the 10 min reaction, add 1 μL of 0.5 M EDTA, mix by pipetting and separate supernatant and bead fraction using a magnetic rack (keep both supernatant and bead fraction).
14. Ethanol precipitate RNAs in the supernatant fraction.
  a. Add 1 μL of 15 mg/mL GlycoBlue to the supernatant fraction.
  b. Add 250 μL 300 mM NaCl.
  c. Add 750 μL 100% ethanol.
  d. Mix thoroughly and freeze at −20°C for at least 2 h.
    ***Note:*** Freezing overnight increases precipitation efficiency.
  e. Spin at 18000 *g* at 4°C for 30 min.
  f. Discard supernatant and add 800 μL 70% ethanol to wash the pellet.
  g. Spin at 18000 *g* at 4°C for 10 min and discard supernatant.
  h. Briefly spin at 18000 *g* at 4°C for 1 min and discard the remaining supernatant.
  i. Dry RNA pellets for 10 min at room temperature.
  j. Resuspend the pellet in 8 μL urea loading buffer.
    **CRITICAL:** The RNA pellet is tiny, so be careful when aspirating the supernatant.
15. For the RNAs in the bead fraction, resuspend the dry beads with 8 μL urea loading buffer.
16. Boil the RNAs from supernatant and the bead fraction at 95°C for 5 min.
17. Analyze the samples (all 8 μL) by 6% TBE_urea PAGE gel electrophoresis and visualize 5′-end fluorescently labeled RNA with a Typhoon imager (place the gel directly onto the imager, then scan the gel). Load fluorescently labeled RNA marker as size marker.

***Note:*** The amount of fluorescently labeled RNA marker should be determined experimentally based on the signal strength of the marker compared to the samples.

## Expected outcomes

**Figure 4:**
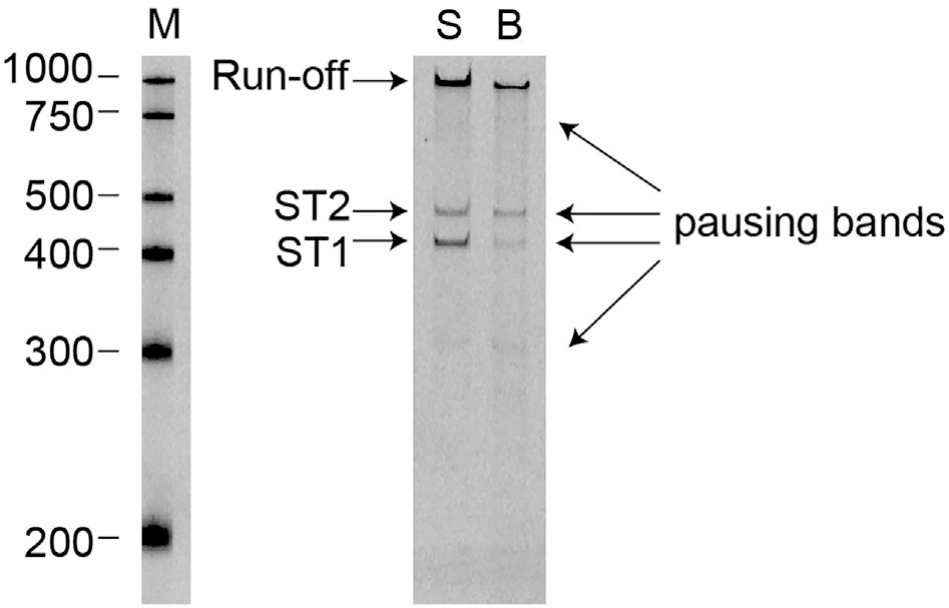
RNAPII spontaneous termination *in vitro* 5′-end FAM-labeled transcripts from supernatant (S) and bead fraction (B) visualized after denaturing 6% TBE-Urea PAGE. ST, spontaneous termination; M, size marker.

For RNAPII *in vitro* transcription, we expect a 1 kb run-off band in both the supernatant and the bead fractions (Figure 4). We would also expect to observe several intermediate bands in the bead fraction, which represent RNA produced due to RNAPII pausing during elongation. Most importantly, we observe intermediate bands in the supernatant fraction when using a CYC1 terminator as the DNA template. Those bands indicate the location where spontaneous termination (ST1 and ST2) takes place.

## Limitations

A limitation of this method is the relatively large amount of purified RNAPII required. We performed *in vitro* transcription experiments with *S. cerevisiae* RNAPII. Unfortunately, mammalian RNAPII is difficult to purify in high quantity, but it is possible to perform these assays with mammalian RNAPII as well.

## Troubleshooting

### Problem 1

Degraded RNAs: If you observe smeared RNA or no RNA in the supernatant fraction, it is most likely due to RNA degradation. Related to step 5.

### Potential solution

• RNase contamination in the reaction can be largely eliminated by washing extensively with TB_1K and TB_hep.
• Use RNase free tubes and filter tips.

### Problem 2

No transcription or no RNA detected. Related to step 2.

### Potential solution

• This issue might be due to problems with assembling the elongation complex. Try optimizing the assembly of the elongation complex, for example, by using more RNAPII.

### Problem 3

Short RNAs, but no long RNA, detected. Related to step 8.

### Potential solution

• This issue might be related to problems with the ligation step. Try optimizing the ligation of the elongation complex to the long transcription template. Consider troubleshooting specific steps, such as BsaI digestion, BamHI clean-up, or the T4 DNA ligase step.

## Resource availability

### Lead contact

Further information and requests for resources and reagents should be directed to and will be fulfilled by the lead contact, Jesper Q. Svejstrup (jsvejstrup@sund.ku.dk).

### Technical contact

Further information and technical questions on the protocol should be directed to and will be answered by the technical contact, Zhong Han (zhonghan@sund.ku.dk).

### Materials availability

This study did not generate new unique reagents.

### Data and code availability

All data reported in this paper will be shared by the lead contact upon request. This paper does not report original code. Any additional information required to reanalyze the data shown here is available from the lead contact upon request.

## Acknowledgments

This work was supported by a Laureate grant from the Novo Nordisk Foundation (NNF19OC0055875) and a Center of Excellence grant (DNRF166) from the Danish National Research Foundation to J.Q.S. and an EMBO Fellowship (ALTF 5-2019) to Z.H. We thank the Svejstrup lab members for discussions. Barbara Dirac-Svejstrup is thanked for her helpful feedback on the manuscript.

## Author contributions

Z.H. optimized the protocol conditions and conducted the methods in this article. J.Q.S. supervised the work and provided funding. Z.H. wrote the manuscript and J.Q.S. revised it.

## Declaration of interests

The authors declare no competing interests.
